# Urban-rural disparities in COVID-19 hospitalisations and mortality: A population-based study on national surveillance data from Germany and Italy

**DOI:** 10.1371/journal.pone.0301325

**Published:** 2024-05-02

**Authors:** Simona Bignami-Van Assche, Federico Ferraccioli, Nicola Riccetti, Jaime Gomez-Ramirez, Daniela Ghio, Nikolaos I. Stilianakis

**Affiliations:** 1 Département de Démographie, Université de Montréal, Montréal, Canada; 2 European Commission, Joint Research Centre (JRC), Ispra, Italy; 3 CERC in Migration and Integration, Toronto Metropolitan University, Toronto, Canada; 4 Department of Biometry and Epidemiology, University of Erlangen-Nuremberg, Erlangen, Germany; Center for Primary Care and Public Health: Unisante, SWITZERLAND

## Abstract

**Purpose:**

Recent literature has highlighted the overlapping contribution of demographic characteristics and spatial factors to urban-rural disparities in SARS-CoV-2 transmission and outcomes. Yet the interplay between individual characteristics, hospitalisation, and spatial factors for urban-rural disparities in COVID-19 mortality have received limited attention.

**Methods:**

To fill this gap, we use national surveillance data collected by the European Centre for Disease Prevention and Control and we fit a generalized linear model to estimate the association between COVID-19 mortality and the individuals’ age, sex, hospitalisation status, population density, share of the population over the age of 60, and pandemic wave across urban, intermediate and rural territories.

**Findings:**

We find that in what type of territory individuals live (urban-intermediate-rural) accounts for a significant difference in their probability of dying given SARS-COV-2 infection. Hospitalisation has a large and positive effect on the probability of dying given SARS-CoV-2 infection, but with a gradient across urban, intermediate and rural territories. For those living in rural areas, the risk of dying is lower than in urban areas but only if hospitalisation was not needed; while for those who were hospitalised in rural areas the risk of dying was higher than in urban areas.

**Conclusions:**

Together with individuals’ demographic characteristics (notably age), hospitalisation has the largest effect on urban-rural disparities in COVID-19 mortality net of other individual and regional characteristics, including population density and the share of the population over 60.

## Introduction

Worldwide, the proportion of Corona Virus Disease 2019 (COVID-19) cases among inhabitants has been higher in cities, compared to sub-urban and rural areas [1]. Cities played a key role in the spread of other potentially pandemic diseases caused by viruses of the *Coronaviridae* family (i.e. Severe Acute Respiratory Syndrome [SARS] and Middle East Respiratory Syndrome [MERS]) [2–4]. Cities’ population density and their high level of inter-connectedness were initially considered as the main reasons increasing transmission of Severe Acute Respiratory Syndrome Corona Virus 2 (SARS-CoV-2) in urban areas [5–7]. However, further research has shown that these urban features are neither isolated nor static factors contributing to cities’ vulnerability to COVID-19 [8].

Recent literature has highlighted the overlapping contribution of demographic characteristics and spatial factors to urban-rural disparities in SARS-CoV-2 transmission and outcomes. In the United States, studies have shown that, together with population density, the socioeconomic characteristics of affected communities such as crowded and poor living conditions have been key factors for COVID-19 spatial spread [9] and mortality patterns [10–13]. This understanding has been crucial to design more efficient health policies and implement targeted interventions such as social distancing during the pandemic [14]. In Europe, several studies have highlighted how, together with demographic characteristics, the geographical and institutional features at the local, regional, and national levels (e.g., population density, mobility, urban scales, active physicians rate, health accessibility, interregional quality) have affected COVID-19 death rates [15] and excess mortality [16–20]. In the Netherlands, Boterman [21] has identified how spatial patterns of COVID-19 infections and hospitalisations have differed in time due to the evolving interplay of population density and public health measures and compliance. His findings provide evidence that pandemic response should have been grounded in a deeper understanding of urban-rural disparities in SARS-CoV-2 transmission and outcomes.

The main limitation of the studies cited above is that they have analysed urban-rural disparities in SARS-CoV-2 transmission and outcomes at the aggregate level, modeling infections, hospitalisations, mortality and spatial factors such as population density as macro-level factors. Nonetheless, in addition to individuals’ physical geographic location, there are social, cultural and environmental differences within the population which may lead to differences in risk factors and hence differences in health outcomes. Individual characteristics are thus key to understand urban-rural disparities in SARS-CoV-2 outcomes, but few studies have been able to disentangle the interplay between spatial and individual factors at the population level due to the lack of appropriate data. An exception is a study in Israel, which modeled sociodemographic disparities in COVID-19 burden by analyzing individual data on confirmed cases, hospitalisations, mortality and vaccinations by ethnic characteristics of localities of residence [22]. By doing so, the authors were able to identify not only the role of place of residence, but especially how ethnic and socioeconomic differences contribute to these differences.

In earlier work, we have contributed to this literature by analysing the demographic characteristics of individuals infected with SARS-CoV-2 and modeling the risk of dying at the individual level net of hospitalisation during the first [23] and second pandemic wave [24]. Nonetheless, we have not explored subnational differences in COVID-19-related mortality and hospitalisation. This paper aims to fill this gap by expanding on our earlier analyses of population-based surveillance data on laboratory confirmed COVID-19 cases and outcomes in Germany and Italy released by the European Centre for Disease Prevention and Control (ECDC). The purpose of this study is twofold. Our first objective is to map COVID-19 hospitalisations and mortality across urban, intermediate and rural territories as defined by the European Statistical Office [25]. Our second objective is to explore the association between hospitalisation and urban-rural disparities in COVID-19 mortality after adjusting for individual characteristics (age and sex) as well as spatial factors (population density and the share of the population over the age of 60), and pandemic wave. By addressing these two objectives, our results will help to better understand the role of place of residence for adverse health outcomes during the COVID-19 pandemic, thus contributing to current healthcare planning as well as future pandemic preparedness.

## Data

As part of its general mandate of infectious disease surveillance, since February 2020 the ECDC has collected and analysed individual-level information on laboratory-confirmed SARS-CoV-2 infections for European Union Member States (beginning in March 2022, case monitoring for SARS-CoV-2 infections has been integrated into the European Respiratory Virus Surveillance system). Access to these data from the European Surveillance System (Tessy) is restricted and was authorized by ECDC. Specifically, we had access on information about all SARS-CoV-2 infections confirmed between February 2020 and November 2021. This period captures what are generally referred to as the first pandemic wave (February–June 2020); the second pandemic wave, ending with the rise of the Omicron variant (July 2020 –February 2021); and the third pandemic wave (March–November 2021), begun with vaccine rollout and the spread of the Omicron and subsequent variants.

For each SARS-CoV-2 confirmed infection, we were given access to anonymised individual information on age, sex and place of residence as well as hospitalisation status (yes, no, or unknown), admission to intensive care (yes, no, or unknown), and clinical outcome (alive, died, still in treatment or unknown). Fatalities refer to those occurring both in hospital and in the community (including nursing homes). Although the dataset includes 28 European countries, due to the incompleteness of information on clinical outcome and hospitalisation status, the analysis in this paper focuses on Germany and Italy, where information on individuals’ hospitalisation status and clinical outcome (death or survival) is at least 98 percent complete. Information on intensive care is not sufficiently complete even in these two countries to be integrated in the analysis.

In the dataset, individuals’ place of residence is recorded according to EUROSTAT’s Nomenclature of Territorial Units for Statistics (NUTS), which distinguishes territories into three categories: (a) urban areas, where more than 80% of the population lives in urban agglomerations; (b) rural areas, where at least 50% of the population lives in rural agglomerations; and (c) intermediate areas, where between 50% and 80% of the population lives in urban agglomerations [25]. According to EUROSTAT’s nomenclature, NUTS1 represent the national level; NUTS2 the regional level; and NUTS3 the provincial level. The number of NUTS3 and the population size of urban, intermediate, and rural areas in Germany and Italy in 2021 is presented in Table 1. In Germany and Italy, approximately 40% of the population lived in intermediate territories in 2021, which represent roughly half of NUTS3 in both countries. Compared to Germany, in Italy a slightly larger share of the population lived in urban areas (48% vs. 44%, respectively), which represent about one fourth of NUTS3 (28% and 28%, respectively). Correspondingly, the share of the population living in rural areas in Italy was 1.5 times less the corresponding share in Germany (10% vs 16%, respectively). On the contrary, in Germany rural areas represented close to a fourth of NUTS3 (28%). Table 1 also shows that the share of the population over 60 was similar in Germany and Italy, and slightly higher in rural than urban areas is both countries (30% vs 26–28%, respectively).

**Table 1:** Number and percent of NUTS3 and population size of urban, intermediate, and rural territories; population density; and share of the population over 60 in Germany and Italy, 2021.

NUTS	Germany	Italy
***National***		
Number of NUTS3^1^	395	102
Total population ^1^	83,155,031	59,236,213
Population density^2^	540.0 people/km^2^	202.9 people/km^2^
Share of the population over 60^1^	28%	29%
***Urban territories***		
Number (percent) of NUTS3^1^	92 (23%)	29 (28%)
Total (percent) population ^1^	36,231,327 (44%)	28,394,212 (48%)
Population density^2^	1319.7	583.8
Share of the population over 60^1^	26%	28%
***Intermediate territories***		
Number (percent) of NUTS3^1^	193 (49%)	55 (54%)
Total (percent) population ^1^	33,923,880 (40%)	24,677,072 (42%)
Population density^2^	391.0 people/km^2^	179.0 people/km^2^
Share of the population over 60^1^	29%	30%
***Rural territories***		
Number (percent) of NUTS3^1^	110 (28%)	18 (18%)
Total (percent) population ^1^	12,999,824 (16%)	6,164,929 (10%)
Population density^2^	149.3 people/km^2^	92.3 people/km^2^
Share of the population over 60^1^	30%	30%

Sources: ^1^ [26]. ^2^ [27].

## Methodology

We estimated the probability of dying given SARS-CoV-2 infection net of hospitalisation, individual characteristics and spatial factors with a generalized linear model (GLM) with binomial distribution where the outcome is whether the individual infected with SARS-CoV-2 died or not. Our covariates of interest are:

- individual covariates: sex; age group (0−19, 20−29, 30−59, 60−69 and 70+); hospitalisation (yes or no);
- spatial covariates: NUTS3 (place of residence classified by degree of urbanization in urban, intermediate, rural); share of the population over 60 at the NUTS3 level (continuous variable, standardized); and population density at the NUTS3 level (continuous variable, standardised).

We also included fixed effects for country (Germany and Italy) and pandemic wave (Wave 1, from January, 2020 to July, 2020; Wave 2, from August, 2020 to June, 2021; Wave 3, from July, 2021 to November, 2021).

In earlier work, we have shown that mortality differentials across countries result from the interaction of demographic risk factors (notably age and sex) and hospitalization at the individual level [23], and that this relationship has changed over the course of the pandemic in Germany and Italy [24]. In the GLM analysis, we thus included an interaction between hospitalisation, age group, and pandemic wave. To test how differentials in COVID-19 mortality between Germany and Italy were associated with differential access to hospital resources in urban, intermediate and rural territories over the course of the pandemic, we also added to the GLM model interactions between NUTS3, hospitalisation and pandemic wave. Finally, following the existing literature [5–7, 15, 21], we modelled how place of residence (NUTS3) interacts with population density and share of the population over 60 to quantify the differential effect of the spatial factors on the risk of dying given SARS-CoV-2 infection.

A GLM model is appropriate for this study since our outcome variable is dichotomous but our covariates are not normally distributed. The model assumes that the outcome variable follows a binomial distribution with a mean that is a function of the linear combination of the predictors. The fixed effects and interactions included in the model minimize biases due to omitted variables, which may affect the model performance.

We performed uncertainty analysis in the GLM to find the 95% confidence interval for the GLM coefficient estimates. The Akaike information criteria (AIC) was used to test the model’s overall goodness-of-fit. Analyses were conducted using R. Ethics approval was not required for this analysis, given the use of aggregate, anonymised data already approved and in use for surveillance of SARS-CoV-2 infections and their outcome.

## Results

### Mapping COVID-19 mortality and hospitalisations across European urban and rural territories

Table 2 shows that, in Italy, urban and intermediate territories had a slightly higher share of SARS-CoV-2 infections (49%) and COVID-19 deaths (48%) than they did in Germany (42% and 39%, respectively) between February 2020 and November 2021. The reverse is true for rural territories where, in Italy, we find approximately half the share of national cases and deaths (9% and 10%, respectively) compared to Germany (18% and 19%, respectively), but almost half more in the share of hospitalisations (25% in Italy and 17% in Germany). Overall, the largest proportion of hospitalisations among laboratory confirmed SARS-CoV-2 infections for Germany and Italy are found in urban and intermediate territories (Table 2). Urban-rural disparities in hospitalisations were larger in Germany (44% in urban areas compared to 17% in rural areas) than in Italy (39% in urban areas compared to 25% in rural areas), whereas the reverse is true for urban-rural disparities in lethal SARS-CoV-2 infections (in Germany, 39% in urban areas compared to 19% in rural areas; in Italy, 48% vs. 10%). These disparities have remained almost unchanged over time (see Table in S1 Table).

**Table 2:** Percent of confirmed SARS-CoV-2 infections, hospitalisations and deaths across urban, intermediate, and rural territories in Germany and Italy, February 2020 –November 2021.

NUTS	Germany	Italy
***National***		
Number of SARS-CoV-2 infections	3,557,410	4,665,175
Number of hospitalisations	304,031	530,053
Number of deaths	83,572	130,056
***Urban territories***		
SARS-CoV-2 infections (percent)	42%	49%
Hospitalisations (percent)	44%	39%
Deaths (percent)	39%	48%
***Intermediate territories***		
SARS-CoV-2 infections (percent)	40%	41%
Hospitalisations (percent)	39%	36%
Deaths (percent)	42%	43%
***Rural territories***		
SARS-CoV-2 infections (percent)	18%	9%
Hospitalisations (percent)	17%	25%
Deaths (percent)	19%	10%

Source: Our calculations on ECDC Tessy data.

**Fig 1:**
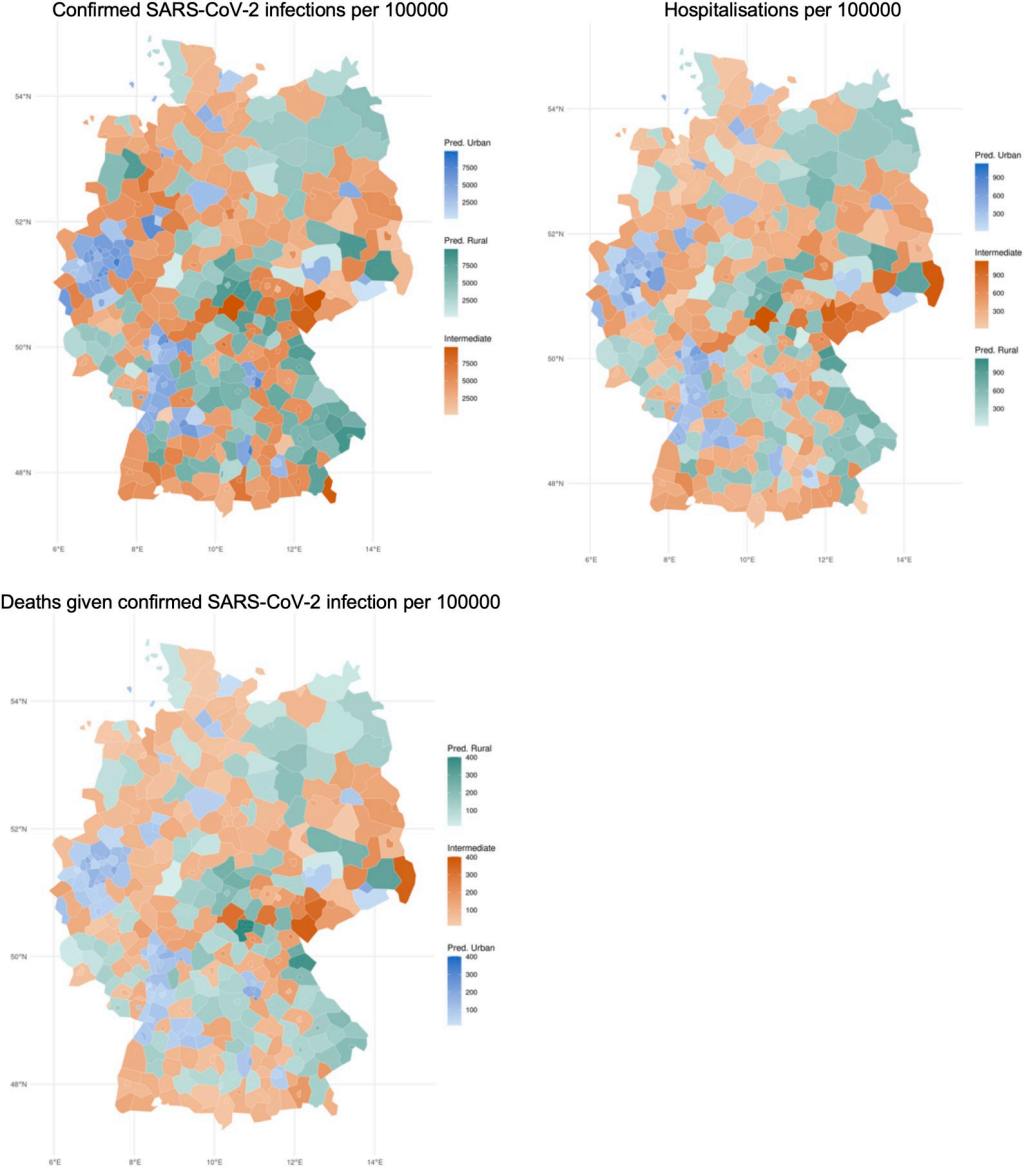
Number of confirmed SARS-CoV-2 infections and number of hospitalisations and deaths given confirmed SARS-CoV-2 infection per 100000 habitants across urban, intermediate and rural territories in Germany, February 2020 –November 2021. Source: EUROSTAT (2022) and Tessy.

Fig 1 visualizes the geographic distribution of the number of deaths and hospitalisations given confirmed SARS-CoV-2 infection per 100,000 habitants across urban, rural and intermediate NUTS3 in Germany between February 2020 and November 2021. Fig 1 shows that the majority of cases per 100,000 inhabitants were located in the central part of Germany, with some other more affected areas in the southeast and north-west. The distribution of hospitalisations and deaths was more localised in the central-eastern region of the country, in particular in the areas of Thuringen, Chemnitz and Dresden.

**Fig 2:**
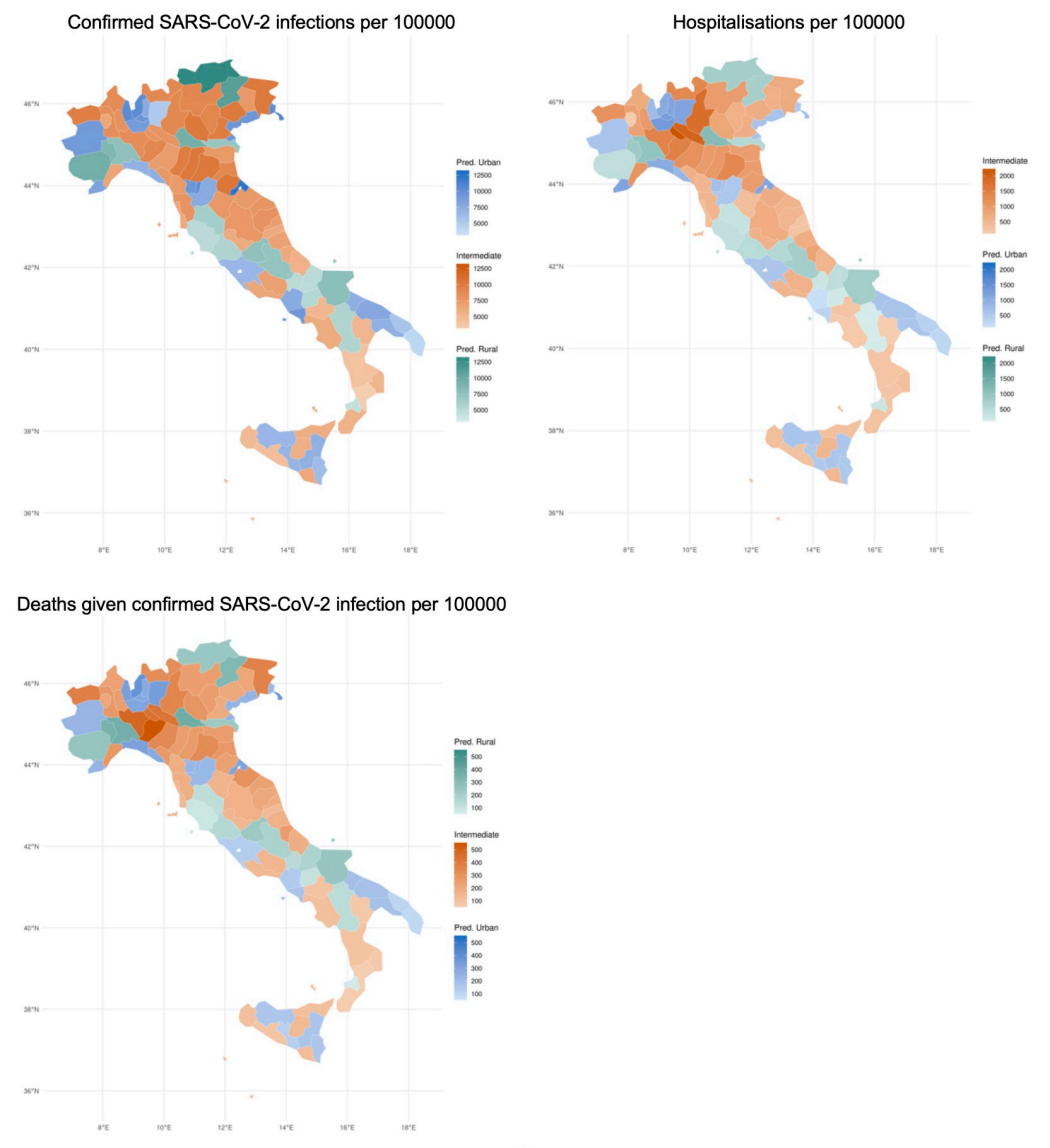
Number of confirmed SARS-CoV-2 infections and number of hospitalisations and deaths given confirmed SARS-CoV-2 infection per 100000 habitants across urban, intermediate and rural territories in Italy, February 2020 –November 2021. Source: EUROSTAT (2022) and Tessy.

In Italy, Fig 2 shows that the highest percentage of cases per capita between February 2020 and November 2021 was found in the northeastern regions, that is, Trentino Alto-Adige, Veneto and Friuli-Venezia Giulia and part of Emilia-Romagna, as well as some of the northern municipalities of Lombardia. The distribution of hospitalisations and deaths was slightly different, however. The largest proportions of hospitalisations per capita was found in Lombardia, and more precisely the provinces of Lodi, Cremona and Bergamo and their surrounding areas. The distribution of deaths was similar, with the majority of fatalities concentrated in the central areas of Lombardia and northwestern part of Emilia-Romagna.

### Urban-rural disparities in COVID-19 mortality risk net of hospitalisation, individual and regional characteristics

Estimated coefficients and standard errors from the GLM model are presented in Table 3. Overall, country differences in COVID-19 mortality are explained by urban-rural differentials in the probability of dying given SARS-CoV-2 infection. In what type of territory individuals live (urban-intermediate-rural) accounts for a significant difference in their probability of dying given SARS-CoV-2 infection, with urban residents particularly disadvantaged compared to rural residents (coeff = −.013, *p* = .005). Country differences in COVID-19 mortality are significant only for intermediate territories, with residents in Italian intermediate territories experiencing a much lower probability of dying than residents in German rural territories (coeff = −.012, *p* = .001). Although the effect is small, population density increases the risk of dying among laboratory confirmed COVID-19 cases overall (coeff = .017, *p* = .001), but it reduces it in both intermediate and urban territories compared to rural ones. We find a similarly small effect for the share of individuals over 60 years of age, which is not significant in intermediate territories compared to rural ones. Finally, consistently with the existing literature, we find that population density and the share of the population over 60 have a small but significant impact on COVID-19 mortality. In line with existing studies, we also find that age (and, to a less extent, sex) has the largest impact on COVID-19 mortality across urban, intermediate and rural territories [8, 10–13].

**Table 3:** Coefficient estimates, standard errors (SE) and p-values estimated from the GLM model predicting the probability of dying given SARS-CoV-2 infection in Germany and Italy.

NUTS	Est. coeff.	SE	Pr(>|z|)
***Regional characteristics***			
*Country* (ref: Germany)	-0.02	0.01	0.118
*Type of territory* (ref: rural)			
Intermediate	-0.09	0.05	0.039
Urban	-0.13	0.05	0.005
Country * Type of territory			
Italy*Intermediate	-0.12	0.02	0.001
Italy*Urban	0.01	0.02	0.506
*Share of the population over 60 years*	0.12	0.02	0.001
*Population density*	0.17	0.06	0.001
*Pandemic wave* (ref: Wave 1)			
Wave2	-1.07	0.02	0.001
Wave3	-2.56	0.05	0.001
***Individual characteristics***			
*Sex* (ref: Women)	0.34	0.01	0.001
*Age* (ref: under 20 years)			
Age 20–29	0.72	0.25	0.001
Age 30–59	3.37	0.21	0.005
Age 60–69	5.57	0.20	0.001
Age 70+	8.17	0.20	0.001
*Hospitalisation* (ref: no)	3.92	0.25	0.001
***Interaction terms***			
Hospitalisation * Age			
Hospitalisation * Age 20–29	-0.06	0.31	0.842
Hospitalisation * Age 30–59	-0.68	0.25	0.007
Hospitalisation * Age 60–69	-1.55	0.25	0.001
Hospitalisation * Age 70+	-2.95	0.25	0.001
Hospitalisation * Wave			
Hospitalisation * Wave2	0.88	0.01	0.001
Hospitalisation * Wave3	1.70	0.03	0.001
Hospitalisation * Type of territory			
Hospitalisation * Intermediate	0.18	0.02	0.001
Hospitalisation * Urban	0.24	0.02	0.001
Type of territory * Wave			
Intermediate * Wave2	-0.14	0.02	0.001
Urban * Wave2	-0.22	0.02	0.001
Intermediate * Wave3	0.02	0.05	0.621
Urban * Wave3	-0.10	0.05	0.026
Type of territory * Population over 60			
Intermediate * Population over 60	0.00	0.01	0.873
Urban * Population over 60	-0.12	0.01	0.001
Type of territory * Population density			
Intermediate * Population density	-0.13	0.06	0.040
Urban * Population density	-0.25	0.06	0.001

Note: The coefficient estimates indicate the average change in the log odds of the outcome variable (that takes value 1 for death and 0 otherwise) associated with a one unit increase in each predictor variable.

**Fig 3:**
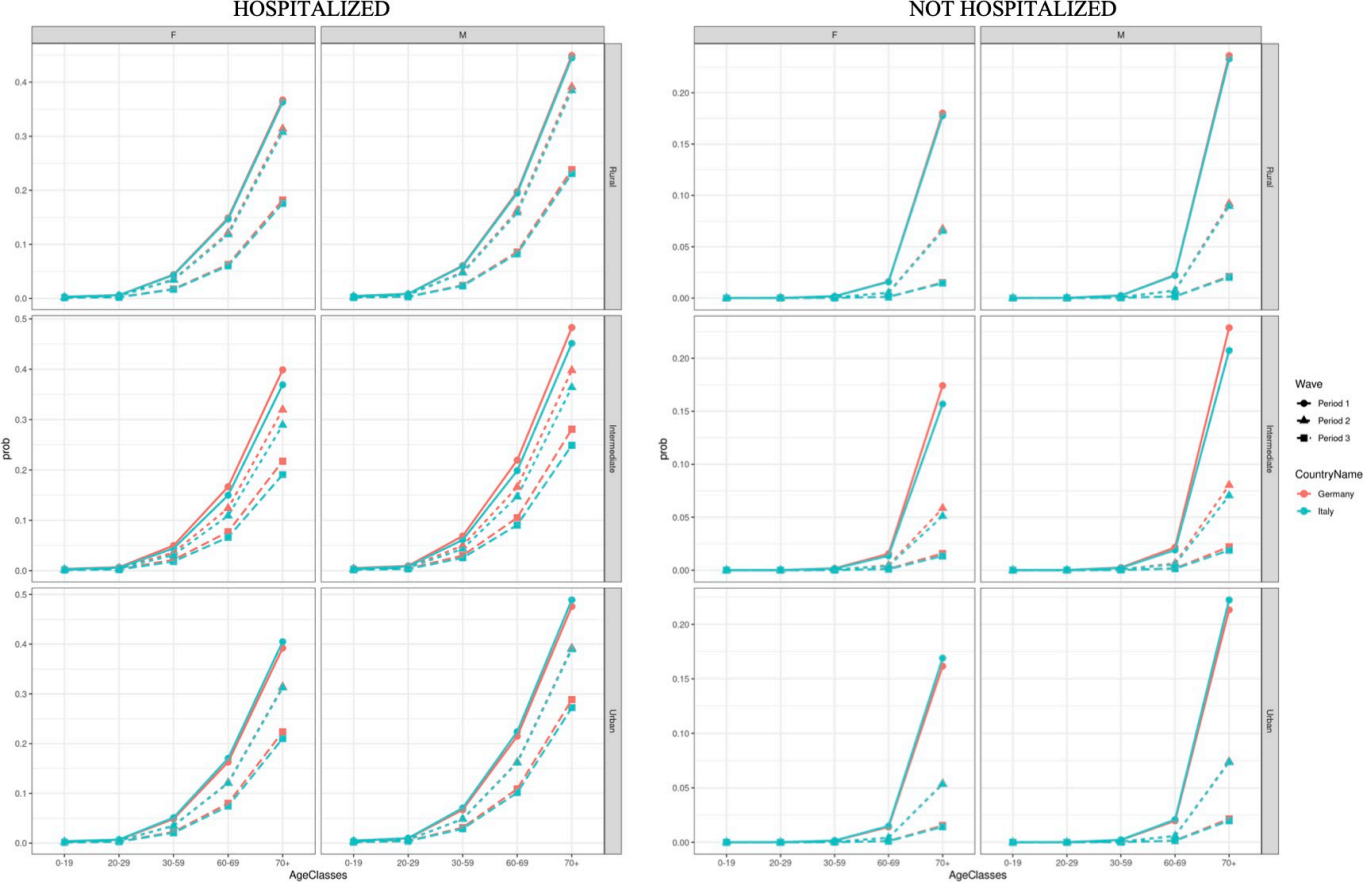
Predicted individual probability of dying for individuals with confirmed SARS-CoV-2 infection who were or were not hospitalised, by country, observation period, age and sex February 2020 –November 2021.

Hospitalisation is a key factor for urban-rural disparities in COVID-19 mortality. To best visualize this association, in Fig 3 the results of the GLM model are presented as predicted probabilities of dying by hospitalization status (hospitalized vs not hospitalized), sex, age group (0−19, 20−29, 30−59, 60−69 and 70+), and for each pandemic wave and country (at the national and urban-rural-intermediate level). Fig 3 shows how hospitalisation has a large and positive effect on the probability of dying given SARS-CoV-2 infection, but with a gradient across urban, intermediate and rural territories. For those living in rural areas, the risk of dying is lower than in urban areas but only if hospitalisation was not needed. Indeed, for those who were hospitalised in rural areas the risk of dying was higher than in urban areas. For individuals living in rural areas, the estimated probability of dying was lower than for the ones living in urban areas. Compared to the first pandemic wave, the association between hospitalisation and death among laboratory confirmed COVID-19 cases increased among waves.

## Conclusion

In this paper, we exploit a large, population-based dataset on SARS-CoV-2 infections and outcomes in Germany and Italy to analyse the interplay between individual characteristics and spatial factors for urban-rural disparities in COVID-19 mortality. Understanding the differential use of hospital resources during the COVID-19 pandemic in urban vis-à-vis rural areas is key for current healthcare planning and future pandemic preparedness, for two main reasons. First, urban-rural differentials in health and mortality were widening in several countries even before the COVID-19 pandemic [28, 29]. Second, population ageing makes these inequalities especially important, since older adults are more affected by insufficient localized health care provision than younger, more mobile populations [30: 102].

The existing literature has not been able to disentangle spatial and individual factors associated with the risk for hospitalisation and death given SARS-CoV-2 infection due to the lack of appropriate data. Existing studies have thus been carried out at the aggregate level [10–13] or have focused on specific countries [21] or regions [31]. Our paper contributes the existing literature by providing the first comparative analysis of urban-rural differentials in COVID-19 mortality between the urban, intermediate and rural areas of two European countries that were particularly affected during the first two waves of the COVID-19 pandemic.

Our data show that the largest proportion of hospitalisations among laboratory confirmed SARS-CoV-2 infections for Germany and Italy between February 2020 and February 2021 were found in urban and intermediate territories. Urban-rural disparities in the proportion of infected cases who were hospitalised were larger in Germany (44% in urban areas compared to 17% in rural areas) than in Italy (39% in urban areas compared to 25% in rural areas), whereas the reverse is true for urban-rural disparities in lethal SARS-CoV-2 infections (in Germany, 39% in urban areas compared to 19% in rural areas; in Italy, 48% vs. 10%). These disparities remained almost unchanged over the first three pandemic waves.

Our main finding is that, together with individuals’ demographic characteristics (notably age), hospitalisation had the largest effect on urban-rural disparities in COVID-19 mortality net of other spatial and individual factors. This result complements our earlier work, which has shown how the demographic patterns of health care utilization have been a key factor of COVID-19-related mortality at the national level in Germany and Italy during the first and second pandemic wave [23, 24]. On the contrary, net of hospitalisation, we find that population density and the share of the population over 60 had a small, albeit significant, impact on COVID-19 mortality.

The strength of this study is that our large, population-based dataset captures both hospitalisation and death for all individuals with laboratory confirmed COVID-19. It also uses existing EUROSTAT categories for place of residence (NUTS1 and NUT3), enabling appropriate comparisons between Germany and Italy. However, we lack information on a number of individual and contextual variables that are known to be associated with the risk of dying because of COVID-19, and may thus bias the GLM model estimates. At the individual level, this is the case for ethnicity, socioeconomic status and comorbidities as well as household living arrangements. Luxenburg et al. [22] found a strong gradient in the need for hospitalisation by ethnicity and socioeconomic status in Israel, which importantly affected geographical disparities in COVID-19 death rates over four pandemic waves. Extensive research has documented the role of individual comorbidities for COVID-19 mortality risk, but only for patients who were hospitalized in specific countries [32] or regions [31], or without considering geographic disparities altogether. In Italy, Basellini and Camarda [16] have found that the degree of intergenerational co-residence is a more important predictor of COVID-19 mortality at the regional level than population density and the share of the population who are older or have at least one chronic disease. At the NUTS3 level, we also did not have information on the availability of hospital resources such as the number of intensive care beds–which have all been found to be significantly associated with COVID-19 mortality in aggregate studies [16].

Lastly, a limitation of our study is that laboratory-confirmed cases of COVID-19 depend on testing criteria that kept changing over the first year of the pandemic. The COVID-19 pandemic has shown how continuous monitoring and timely release of information on the demographic, epidemiological, and socioeconomic characteristics of infections is needed to correctly estimate the probability of hospitalisation and death. Population-based studies able to integrate all these dimensions are needed for future pandemic preparedness.

## Supporting information

S1 Table Percent of confirmed SARS-CoV-2 infections, hospitalisations and deaths across urban, intermediate, and rural territories in Germany during the first, second and third pandemic waves, February 2020 –November 2021. (DOCX)
