# Supplementary material for: Urban-rural disparities in COVID-19 hospitalisations and mortality: A population-based study on national surveillance data from Germany and Italy
Source: PLoS One. 2024 May 2;19(5):e0301325. doi: 10.1371/journal.pone.0301325 (PMC11065260; doi:10.1371/journal.pone.0301325)
Supplement: S1 Table — (DOCX) [file pone.0301325.s001.docx]

**Supporting information**

**S1 Table**. **Percent of confirmed SARS-CoV-2 infections, hospitalisations and deaths across urban, intermediate, and rural territories in Germany during the first, second and third pandemic waves, February 2020 – November 2021**

|  | GERMANY | | | ITALY | | |
| --- | --- | --- | --- | --- | --- | --- |
| NUTS | Wave 1 | Wave 2 | Wave 3 | Wave 1 | Wave 2 | Wave 3 |
| ***National*** |  |  |  |  |  |  |
| Number of infections | 180,699 | 2,711,735 | 664,976 | 244,012 | 3,917,461 | 503,702 |
| Number of hospitalisations | 30,544 | 236,037 | 37,450 | 88,363 | 315,324 | 26,754 |
| Number of deaths | 8,956 | 70,430 | 4,186 | 35,786 | 90,507 | 3,763 |
| ***Urban territories*** |  |  |  |  |  |  |
| SARS-CoV-2 infections (%) | 43% | 42% | 44% | 44% | 50% | 47% |
| Hospitalisations (%) | 43% | 44% | 48% | 45% | 49% | 48% |
| Deaths (%) | 37% | 39% | 43% | 46% | 48% | 51% |
| ***Intermediate territories*** |  |  |  |  |  |  |
| SARS-CoV-2 infections (%) | 41% | 40% | 40% | 47% | 40% | 45% |
| Hospitalisations (%) | 40% | 39% | 37% | 49% | 42% | 45% |
| Deaths (%) | 43% | 41% | 41% | 46% | 41% | 43% |
| ***Rural territories*** |  |  |  |  |  |  |
| SARS-CoV-2 infections (%) | 16% | 18% | 16% | 9% | 10% | 8% |
| Hospitalisations (%) | 18% | 17% | 15% | 6% | 9% | 7% |
| Deaths (%) | 20% | 20% | 16% | 8% | 11% | 7% |

Source: Our calculations from ECDC Tessy data.
